# Application of Proteomics in the Discovery of Radiosensitive Cancer Biomarkers

**DOI:** 10.3389/fonc.2022.852791

**Published:** 2022-02-23

**Authors:** Hui Luo, Hong Ge

**Affiliations:** Department of Radiation Oncology, The Affiliated Cancer Hospital of Zhengzhou University, Zhengzhou, China

**Keywords:** tumor, radiosensitive biomarker, proteomic, application, review

## Abstract

Radiation therapy remains an important component of cancer treatment. Gene-encoded proteins were the actual executors of cellular functions. Proteomic was a novel technology that can systematically analysis protein composition and measure their levels of change, this was a high throughput method, and were the import tools in the post genomic era. In recent years, rapid progress of proteomic have been made in the study of cancer mechanism, diagnosis, and treatment. This article elaborates current advances and future directions of proteomics in the discovery of radiosensitive cancer biomarkers.

## 1 Introduction

Radiation therapy is a highly targeted treatment accurately suppressing the tumor with wide ranging application, and contributed to approximately 40% of all cancer cures across the world (1). This treatment modality is also very effective for other cancer related problems including pain, tumor bleeding, and acute superior vena cava syndrome (2–4). Despite the great benefit, the existence of radiation resistance leads to local recurrence, distant metastasis, and poor survival, this is really a key challenge in cancer treatment (5). With the development of precision medicine in clinic practice, there was an urgent need to screen a number of biomarkers for patient’s stratification and thus develop appropriate treatment strategies (6). Indeed, investigators were dedicated to identify potential radiosensitivity biomarkers to perform tailored radiation therapy for cancer patients (7, 8). Big data was the basis for the realization of precision medicine (9). After the completion of human genome sequencing, the “post-genome” era has arrived (10). Genes cannot perform the functions by themselves, and their functions need to be performed directly through transcription and translation to proteins (11).

Protein expression can be influenced by environmental conditions, nutritional status, and therapeutic modalities. Protein expression can directly reflect different physiological or pathological processes in the organism (12). Proteomic was an advanced systemic biology research method in the “post-genomic” era, it was studied by large-scale screening and identification of protein expression in body fluids, tissues, cells or organisms under various conditions, and protein-protein interactions will be well recognized (13). Mass spectrometry (MS) has become an important tool for large-scale protein identification in proteomics (14). Currently, research of proteomics mainly focused on basic medicine, health screening, disease prevention, prognosis prediction, patient stratification, and the identification of new targets (15–18).

**Figure 1:**
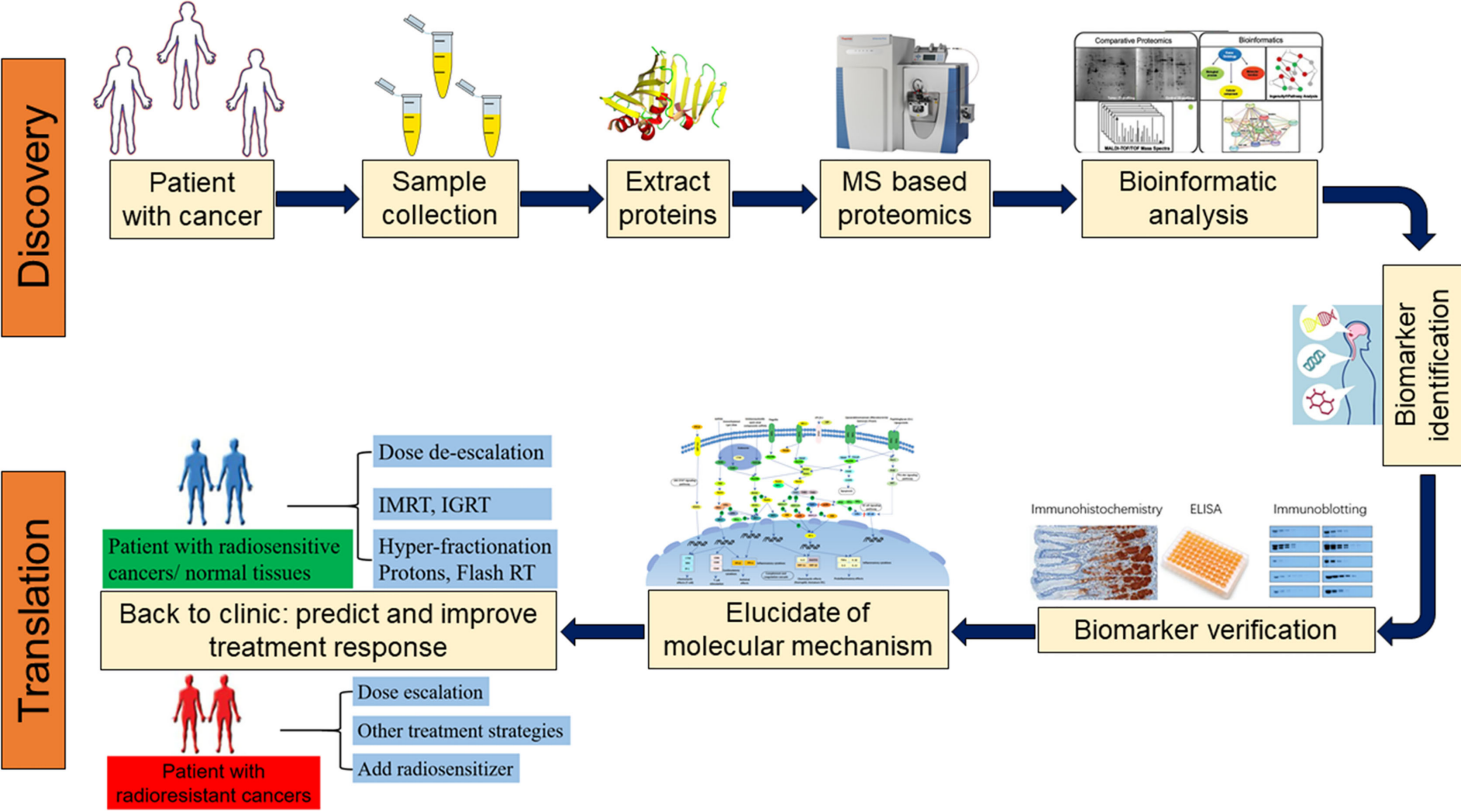
Proteomics approaches for the discovery, validation, and clinical application of biomarkers. (MS, mass spectrometer; IGRT, Image-guided radiation therapy; IMRT, Intensity-modulated radiation therapy).

Since proteins can directly reflect cellular biological processes and were dynamic changed in real time, the application of proteomics in precision medicine has attracted much attention (19, 20). In recent years, researchers attempt to analyze protein expression in tumor tissue and plasma during radiation therapy by proteomics technology, this will be facilitating the detection of proteins that play a key role during the course of radiotherapy and biomarkers that can predict radiotherapy sensitivity in the earlier phase (21, 22). With the development of proteomics, tumor radiation therapy has entered a new era in the context of precision medicine (23). In the present analysis, we reviewed the research progress of proteomics in screening tumor radiotherapy sensitivity markers (**Figure 1**).

## 2 Application of Proteomics to Identify Predictive Biomarkers of Radiosensivity

**Figure 2:**
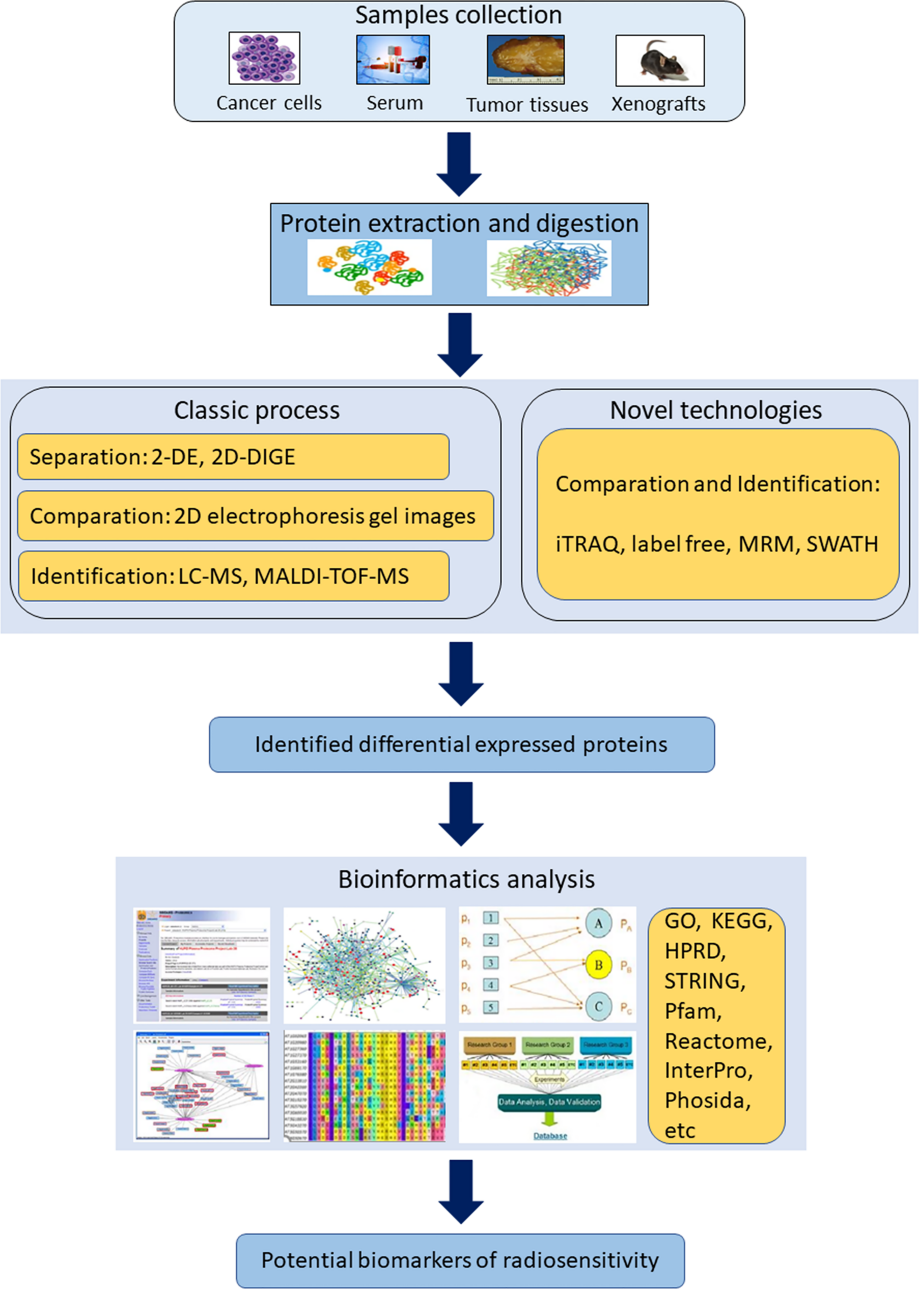
Scheme of the workflow and methodological strategies for proteomics application in identifying radiosensitivity biomarkers. (2-DE, two-dimensional gel electrophoresis; 2D-DIGE, two-dimensional difference gel electrophoresis; GO, The Gene Ontology; HPRD, human protein reference database; iTRAQ, isobaric tags for relative and absolute quantitation; KEGG, Kyoto Encyclopedia of Genes and Genomes; MALDI-MS, matrix-assisted laser desorption ionization mass spectrometer; MRM, multiple reaction monitoring; SWATH, sequential window acquisition of all theoretical fragment ion spectra).

Technological advances in MS have driven an increased capability for rapid, accurate and effective proteomic discovery. To date, several proteomics techniques have been applied to identify potential biomarkers of radiosensitivity (**Figure 2**).

### 2.1 Matrix-Assisted Laser Desorption/Ionization Time-of-Flight Mass Spectrometry (MALDI-TOF-MS)

MALDI-TOF-MS is an approach in proteomics to identify proteins from simple mixtures frequently combined with two-dimensional gel electrophoresis (24). Relatively less intense sample preparation was required for this technique; besides, peaks in a spectrum were typically used as indications of peptide content. However, reproducibility of outcomes may be a problem because of this technique is sensitivity to contaminants from salts. MALDI-TOF-MS may be replaced with the emerging of advanced MS techniques.

### 2.2 Isobaric Tags for Relative and Absolute Quantification (iTRAQ)

iTRAQ technology is a shotgun-based quantization method that uses isobaric reagents to label all primary amines of peptides and proteins (25). The concentration of potential radiosensitivity biomarkers in cancers can easily be quantified using iTRAQ-based proteomics because of the advantages including less time consuming and decreased variation (26). Notably, this technique is expensive, sensitive to contamination from salts, and the variability will arise if the enzymatic digestion is inefficient.

### 2.3 Liquid Chromatography With Tandem Mass Spectrometry (LC-MS/MS)

LC-MS/MS is an analytical chemistry technique that includes physical separation of the analytes in liquid samples and followed by their mass-based identification (27). This technology contributed to the determination of the accurate mass, putative formula, and elucidate the structure of the analyte with relatively small amounts of samples. Therefore, it has been widely used for the analysis of both small molecules and large protein molecules. Nevertheless, the instruments are extremely costly. Sample preparation is important for the extraction of target compounds from complex samples such as serum; otherwise, the system may be contaminated and resulted in a decreased sensitivity (28). Additionally, it is time consuming because of the large amount of information acquired from LC-MS/MS.

### 2.4 Multiple Reaction Monitoring (MRM)

MRM, also known as Selective Reaction Monitoring (SRM) is a technique based on triple quadrupole mass spectrometer and use the signal of selected tandem mass spectrometry fragment ions for further quantification (29). This method can eliminate most of the non-target detection and improve the detection sensitivity of the target proteins in complex samples, the results are reproducible. Meanwhile, MRM could be used for biomarker validation and has the potential to serve as a complementary tool to traditional methods including western blot, immunohistochemistry, and enzyme-linked immunosorbent assay (30). Despite these advantages, MRM may not suitable for proteins that are too short and variable, such as immunoglobulin. For the selected peptides from same target proteins, were observed, incomplete trypsin digestion of parental ions may lead to different quantitation outcomes (31).

### 2.5 Sequential Window Acquisition of All Theoretical Mass Spectra (SWATH-MS)

SWATH-MS is an emerging proteomic platform based on data independent acquisition (DIA) principle for label free quantification (32). It provides a complete and permanent information of all fragment ions in a sample, moreover, a spectral library is necessary to analysis the SWATH-MS data. This technology allows quantitative analysis of peptides covering a great amount of proteins with a high quantitative accuracy and reproducibility. The current disadvantage of SWATH-MS is that the peptide quantification is still less sensitive compared with MRM (33). SWATH-MS is a promising technique and novel algorithms are needed for identifying cancer radiosensitivity factors.

## 3 Radiosensitivity Biomarkers in Cancers

Approximately 50% of tumors need to be treated with radiation therapy, and how to select eligible patients to receive radiotherapy was really a problem (34). Proteins expression were altered during radiotherapy, the application of proteomics to analyze these differential proteins has the potential to identify potential biomarkers of radiation resistance and further revealing the underlying molecular mechanisms. This will contribute to screen patients that eligible for radiotherapy and reveal novel targets of anticancer therapy. In the current study, the following terms were utilized: “neoplasms”, “proteomic”, “radiotherapy”, we also manually checking reference lists to identify additional relevant studies.

### 3.1 Head and Neck Cancers

Head and neck cancers were the sixth leading cancer by incidence and the eighth major cause of cancer related death across the world, and squamous cell carcinoma was the most common type (35). Intensity-modulated radiotherapy was one of the most effective treatment strategies for head and neck cancers (36). Despite a good survival rates of 40%-50%, some patients still exhibit resistance to radiotherapy and resulted in tumor recurrence (37). Identification of biomarkers related to head and neck cancer radiation resistance would helpful to improve patient survival.

#### 3.1.1 Nasopharyngeal Carcinoma

Radiotherapy with or without chemotherapy is the standard treatment option for nasopharyngeal cancer because of the complex anatomical location of this disease (38). Radiation resistance is a severe obstacle to the successful treatment of nasopharyngeal carcinoma, and biomarkers related to the radiation response is of great importance (39). Wu et al. analyzed the proteomic changes of tumor tissues before and after radiation therapy in patients of nasopharyngeal carcinoma and found that endoplasmic reticulum protein 29 (ERP29), manganese superoxide dismutase (Mn-SOD), heat shock protein 27 (HSP27), and glutathione S-transferase (GST) were significantly upregulated and correlated with radiation resistance; ERp29 was significantly overexpressed in radiation-resistant tumor tissues as verified by immunohistochemistry; the application of small RNA silencing technique to downregulate ERP29 expression enhanced both radiosensitivity and apoptosis in CNE-1 and 6-10B cells (40). This study suggests that ERP29 has the potential to be used as a marker for predicting the efficacy of radiotherapy in nasopharyngeal carcinoma. HSP27 was a chaperone belonged to the small heat shock protein family, the protein involved in the inhibition of apoptotic cell death, regulation of cell proliferation and differentiation (41). The analysis by Zhang et al. also confirmed HSP27 as a biomarker of radiation resistance in nasopharyngeal cancer (42). Zhang et al. performed serum proteins profiles using LC-MS/MS and found secreted protein acidic and cysteine rich (SPARC), serpin family D member 1S (ERPIND1), complement C4B, peptidylprolyl Isomerase B (PPIB), and family with sequence similarity 173-member A (FAM173A) were associated with radiation resistance (43). To date, the role of SPARC, SERPIND1, and FAM173A in cancer remains unclear. Complement C4B was a component of the classical activation pathway, dysregulated complement expression within the tumor microenvironment was capable of suppressing antitumor immunity and resulted in poor clinical outcomes (44). PPIB was a cyclosporine-binding protein that participated to regulate cyclosporine A-mediated immunosuppression, further investigation revealed PPIB conferred radiation resistance in head and neck cancer *via* facilitating DNA repair (45).

In addition, Feng et al. established a radiation-resistant cell line CNE2-RR from fractionated radiotherapy irradiated nasopharyngeal carcinoma CNE2 cell line, and proteomic analysis identified 34 cases of differential proteins; after validated by 90 clinical tissue specimens of nasopharyngeal carcinoma, they identified that 14-3-3σ and maspin were significantly downregulated, conversely, GRP78 and Mn-SOD were significantly upregulated; cellular studies confirmed that upregulation of 14-3-3σ could effectively improve the radiation sensitivity of CNE2-RR cell lines (46). GRP78 was a multifunctional protein folding chaperone in endoplasmic reticulum (ER), overexpression of GRP78 was associated with poor survival; suppressing GRP78 enhanced the efficacy of radiation therapy in cancer models (47). Li et al. compared CNE2-RR cell lines with CNE2 cell lines with MALDI-TOF-MS and uncovered 16 cases of proteins related to radiation sensitivity in CNE2-RR cell lines; among them, Nm23H1 was significantly overexpressed and annexin A3 was significantly downregulated; however, the final results were not validated (48). With the same cell lines, another study reported fibrillin-2, CD166, sulfhydryl oxidase 1 and cofilin-2 as biomarkers of radiosensitivity using iTRAQ technique, further analysis revealed the 4 proteins involved in cell adhesion, migration and invasion (49). Similarly, Li et al. analyzed the CNE2-RR cell lines using LC-MS/MS and found MAPK15 was involved in radiation resistance, the potential mechanisms were MAPK15 attenuated reactive oxygen species accumulation and promoted DNA damage repair (39). Accordingly, the above studies suggested that the results of different proteomic techniques were not identical, and further analyses were required in improving the homogeneity of these proteomic techniques.

#### 3.1.2 Other Types of Head and Neck Cancers

Comparative analysis of protein profiles of radiation resistant cell lines QL1, SCC15 and SCC25 verified that Non-metastatic protein 23 H1 (Nm23-H1) was highly expressed and could be used as a marker of radiation resistance (50). Nm23-H1 served as a housekeeping enzyme and involved in suppressing cancer cell metastasis, the activation of Nm23-H1 was capable of inhibiting cancer metastasis *via* redox regulation (51). Comparative proteomic analysis of head and neck squamous carcinoma cell lines FaDu and SCC25 with radiation-resistant cell lines FaDu-RR and SCC25-RR showed that Ras-related C3 botulinum toxin substrate 1 (Rac1) could be used as a new therapeutic target (52). Rac1 is a cytoskeleton involved in cell adhension, morphology, and movement; overexpression of Rac1 is associated with unfavorable survival in different type of cancer (53).

Interestingly, Matsukawa et al. performed a proteomic analysis of tumor tissues from 18 patients with locally advanced oral squamous carcinoma treated with neoadjuvant radiotherapy, galactose lectin 7 was identified and validated as a predictor of radiation resistance with a sensitivity of 96%, a specificity of 39.5%, in the meantime, the 5-year disease-specific survival rate was 75.2% in the galactose lectin 7 low expression group and 100% for the high expression group (54). Further analysis also confirmed that the invasive ability was decreased in galactose lectin 7 overexpressed cells; however, the invasive ability was not significantly altered after knockdown of galactose lectin 7. Therefore, further investigation was necessary for galactose lectin 7 in predicting radiation resistance. Lin et al. analyzed three head and neck tumor cell lines, KB, SAS, and OECM1, and found that five proteins including Heat shock protein 96 (Gp96), Glucose regulatory protein 78 (GRP78), Heat shock protein 60 (HSP60), Ras-related protein Rab-40B (Rab40B) and Growth/differentiation factor-15 (GDF-15) was upregulated, while membrane-linked protein V was downregulation and was associated with radiation resistance; moreover, after silencing of Gp96, tumor cell growth was stunted, clonogenesis was diminished, and the proportion of cells in G2/M phase was increased; in the Gp96-silenced mouse transplant tumor model, the tumor size was significantly reduced when compared with normal mouse after receiving radiotherapy (55). Most of the biomarkers discovered in Lin et al’s study were involved in the regulation of ER stress, and ER stress has been depicted as a hallmark of cancer (56). Our previous analysis also indicated ER stress is associated with radiation resistance (57). Proteomic analysis of the laryngeal cancer cell line HEp-2 and the radiation-resistant cell line HEp-2-RR showed that Chloride Intracellular Channel 1 (CLIC1) induces radiation resistance by inhibiting the production of reactive oxygen species (58). These studies suggest that a number of proteins were involved in the regulation of radiation sensitivity and can be used to predict radiotherapy efficacy and facilitate the development of novel strategies in anticancer therapy.

### 3.2 Thoracic Cancers

In recent years, the incidence of thoracic cancers including esophageal cancer, breast cancer, and lung cancer, were gradually increased across the world (59). Radiotherapy plays a key role in thoracic cancer patients that were not suitable for surgery or early stage non-small cell lung cancer, however, radiation resistance was a common cause of treatment failure (60, 61). Thereby, it was important to identify biomarkers of radiation resistance for each type of chest tumors.

#### 3.2.1 Esophageal Cancer

With the application of two-dimensional protein liquid chromatography system and linear trap quadruple mass spectrometer, Cui et al. analyzed the serum proteomic profiles before and after radiotherapy of Kazakh esophageal cancer patients in Xinjiang and found 21 significant different expressed protein spots peaks; Clusterin (CLU) was detected as a radiation resistant biomarker, and Apolipoprotein A1 (APOA1) was discovered as a serum biomarker of esophageal squamous cell carcinoma (62). Based on next-generation MS, Maher et al. analyzed plasma protein expression changes before, during, and after radiotherapy in 31 esophageal cancer patients treated with neoadjuvant radiotherapy, final results indicated that complements C3a and C4a could be utilized in predicting the efficacy of neoadjuvant radiotherapy with a sensitivity of 78.6% and a specificity of 83.3% (21). Since then, the molecular mechanisms of allergenic toxins in predicting radiotherapy efficacy have been elaborated. Surace et al. found that radiotherapy induced tumor cell death, activated the complement system, and produced complement C3a and C5a, both C3a and C5a were concentrated in tumor tissue in the short term and induced specific anti-tumor immune responses; besides, glucocorticoids suppressed complement activation and decreased the efficacy of radiotherapy, therefore, these drugs should be administered with caution in cancer patients during radiotherapy (63). The study further confirmed that the application of immunosuppressants, such as dexamethasone, would lead to reduced treatment efficacy during radiotherapy. Proteomic analysis of esophageal cancer reveals a close relationship between radiotherapy and anti-tumor immune response, which may provide novel ideas for clinical studies of radiotherapy and immune checkpoint inhibitors.

#### 3.2.2 Breast Cancer

Yang et al. used X-ray to treat breast cancer MDA- MB-231 cell line, and eight differential proteins were identified by MS as the following: Heat shock protein 70 (HSP70), Inosine Monophosphate Dehydrogenase 2 (IMPDH2), Eukaryotic Translation Initiation Factor 4H (EIF4H), Glyceraldehyde 3-phosphate dehydrogenase (GAPDH), Vimentin (VIM), Tubulin Alpha 1b (TUBA1B), Tubulin Alpha 8 (TUBA8) and GRP78 (64). Kim et al. investigated protein expression profiles of breast cancer MDA- MB-231 BC cell treated with single or fractionated radiotherapy and found that several tumor-derived factors including CTSD (Cathepsin D), Gelsolin (GSN), and Mannose Receptor C Type 2 (MRC2) were upregulated, the results also suggested that these factors were involved in regulating the immune response during radiotherapy (65). These screened proteins may be involved in regulating breast cancer cell radiosensitivity and further studies were needed. Comparative proteomic analysis of the breast cancer radiation-sensitive cell line MCF+FIS4 and the radiation-resistant cell line MCF+FIR3 revealed that peroxidase II (PrxII) was significantly increased in the MCF+FIR3 cell line, and silencing of PrxII increased the radiation sensitivity of breast cancer cells, conversely, overexpressing PrxII in breast cancer cells resulted in radiation resistance; further exploration showed that reactive oxygen species were essential for breast cancer radiation resistance and stress-induced overexpression of PrxII protects breast cancer cells from radiation-induced oxidative damage and thus attenuates radiation sensitivity (66). Serum based LC–ESI-MS/MS analysis confirmed oxidative stress response, acute phase response, and coagulation properties were hallmarks of radiosensitivity in breast cancer (67). However, it was worth noticing that the key molecular pathways involved in radiosensitivity is somehow influenced by the radiation doses (68). Guo et al. analyzed the protein kinase profiles of MCF-7 cell line and its cognate radiation-resistant cell line MCF-7/C6 and validated the results showed that checkpoint kinase 1 (CHK1), cell cycle protein-dependent kinases 1 and 2 (CDK1 and CDK2) were significantly upregulated in the radiation resistant cell lines; this suggests that both DNA repair and cell cycle were involved in regulating breast cancer radiation resistance (69).

To detect the homogeneity and heterogeneity between different proteomics techniques, Laura et al. analyzed breast cancer radiation resistant cell lines (MCF7-RR, MDA-MB-231-RR and T47D-RR) with their cognate radiation therapy sensitive cell lines using three different proteomics techniques: the two-dimensional gel electrophoresis in combination with MS, liquid chromatography coupled with tandem mass spectrometry, and quantitative proteomics using iTRAQ technique; the resules verified that 26S proteasome and GRP78 were significantly down-regulated in all radiation-resistant cell lines, in addition, very few overlapping proteins were identified by the three techniques, which indicating the different advantages with different proteomics techniques (70). Over the past decades, GRP78 has been recognized as a target in anticancer strategies (71). Several compounds and antibodies targeting GRP78 have been investigated in various type of cancers (47, 72, 73).

Although the aforementioned studies uncovered several potential radiation-resistant biomarkers, their clinical significance has not been well investigated. Pietrowska et al. analyzed the protein expression profiles of plasmas from breast cancer patients treated with surgery and adjuvant radiotherapy, in the surgery alone group, there was insignificant changes in protein expression between preoperative and postoperative serum samples; in patients receiving adjuvant radiotherapy, they detected two serum peptides (registered m/z values 2,184 and 5,403 Da) were significantly downregulated (74). This study confirmed that radiotherapy can modulating protein expression in the plasma of cancer patients, however, further bioinformatics analysis was needed to identify potential biomarkers of radiation resistance.

#### 3.2.3 Non-Small Cell Lung Cancer

Yun et al. established a radiation resistant H460 (RR-H460) cell line that displayed cancer stem like cell phenotypes from parental radiosensitive H460 non-small cell lung cancer cells, proteomic analyses of the two cell lines by LC-MS/MS revealed VIM, GRP78, fatty acid synthase (FASN), ubiquinol-cytochrome C reductase complex core protein 1 (UQCRC1), plasminogen activator inhibitor 2 (PAI-2), nodal modulator 2 (NOMO2), kinesin light chain 4 (KLC4) and procollagen-lysine 2-oxoglutarate 5-dioxygenase 3 (PLOD3) as radiation resistance biomarkers. Among these identified proteins, VIM, GRP78, FASN, UQCRC1 have been validated in the prediction of radiation resistance (75). Indeed, GRP78 and VIM have been depicted as radiation resistance factors in other type of tumors (46, 65). Walker et al. collected plasma from patients with locally advanced non-small cell lung cancer treated with radical radiotherapy, proteomic analysis revealed that C-reactive protein (CRP), Lipopolysaccharide Binding Protein (LBP), and Leucine Rich Alpha-2-Glycoprotein 1 (LRG1) were significantly altered during radiotherapy, in the squamous lung cancer subgroup, both CRP and LRG1 were significantly elevated in patients with survival <17 months (22). CRP is a predominant protein of the acute phase response; this protein involved in inflammation response and the developing of pro-metastatic tumor microenvironment (76). LRG1 promotes neovascularization *via* regulating TGF-β signaling (77). Radiotherapy induces the expression and release of pro-angiogenic factors, and the combination of LRG1 inhibitors might be a promising strategy in improving the efficacy of radiotherapy and patients’ prognosis (22). A proteomic analysis of non-small cell lung cancer conducted by Huang et al. identified six potential biomarkers in the radiation-resistant group, the results suggested that α1 antitrypsin could be used as a marker of radiation resistance (78). In conclusion, the above analyses suggested that the screening of protentional biomarkers before and after radiotherapy using proteomics technology can not only be used to predict radiotherapy efficacy and patient survival, but also contribute to the design of novel anti-tumor agents.

### 3.3 Abdominopelvic Tumors

Abdominopelvic tumors account for more than half of tumors in the human body, and radiotherapy was extremely valuable in the comprehensive treatment of abdominopelvic tumors. Nevertheless, the existence of radiation resistance lead to cancer recurrence or progression. Screening for biomarkers of radiation resistance was useful in stratifying patients with abdominopelvic tumors and optimal treatment strategies can be conducted.

#### 3.3.1 Rectal Cancer

The protein profiles of rectal cancer tissue of patients before and after radiotherapy were analyzed by MALDI-TOF-MS, both ERp57and Galectin-3 were the main biomarkers identified (79). ERp57 belonged to the protein disulfide isomerase family, this protein conferred immune escape *via* multiple immune regulatory processes (80). Galectin-3 was a member of the lectin family and contributed to tumor microenvironment immunosuppression and immune cell regulation (81). Besides, Chauvin et al. analyzed the protein expression profile of 23 rectal cancer patients treated with neoadjuvant radiotherapy and found that Interferon Induced Protein with Tetratricopeptide Repeats 1 (IFIT1), FAST Kinase Domains 2 (FASTKD2), Phosphatidylinositol-5-Phosphate 4-Kinase Type 2 Beta (PIP4K2B), AT-Rich Interaction Domain 1B (ARID1B), and Solute Carrier Family 25 Member 33 (SLC25A33) were overexpressed in patients achieved complete remission, in the meantime, Caldesmon 1 (CALD1), Carboxypeptidase A3 (CPA3), Beta-1,3-Galactosyltransferase 5 (B3GALT5), Receptor Interacting Serine/Threonine Kinase 1 (RIPK1), and CD117 were upregulated in patients with poor response (82). Among these identified biomarkers, PIP4K2B was a lipid kinase and involved in the regulation of cancer cell proliferation *via* transduced changes in GTP into changes in the levels of the stress regulated phosphoinositide PtdIns5*P* (83). Furthermore, CALD1, a biomarker of cancer associated fibroblasts, was found to be an independent biomarker of poor survival by gene expression profiles of micro-dissected rectal cancer (84). To date, the mechanisms of these altered proteins mediate radiation resistance in rectal tumor cells have not been fully elucidated, and large sample size clinical studies were required. Additionally, it should be noted that the changes of radiosensitivity biomarkers in serum may be tumor stages specific (81).

#### 3.3.2 Prostate Cancer

Skvortsova et al. compared the protein profiles between three prostate cancer radiation therapy-resistant cell lines (PC3-RR, DU145-RR, and LNCaP-RR) and their cognate radiation sensitive cell lines, the result revealed five proteins were significantly altered: NME/NM23 Nucleoside Diphosphate Kinase 1 (NME1), Heat Shock Protein Family A Member 8 (HSPA8), Apurinic/Apyrimidinic Endodeoxyribonuclease 1 (APEX1), and Ras-related protein Rab-11A (RAB11A); APEX1 was an DNA repair enzyme, knockout of APEX1 significantly enhanced radiosensitivity of prostate cancer cells (85). It was worth noting that NME1 served as a metastasis suppressor in cancer cells, this enzyme decreased pEGFR and pAkt expression in a dynamin dependent manner and contributed to metastasis suppression *via* altering tumor endocytic and motility phenotypes (86). Chang et al. analyzed the protein profiles of three prostate cancer radiation-resistant cell lines (PC3, DU145, and LNCaP) and their cognate radiation sensitive cell lines using label-free liquid chromatography tandem mass spectrometry, final results revealed Aldolase, Fructose-Bisphosphate A (ALDOA) involved in the regulation of radiation sensitivity both on prostate cancer cell lines and its transplant tumor models (87). Both aldolase and ALDOA involved in glucose metabolism, these enzymes promoted cancer cell metastasis *via* interacting with γ-actin (88). One of the glycolysis markers, lactate dehydrogenase A has been demonstrated as a biomarker of radiation resistance in a radioresistant prostate cancer xenograft mouse model using LC-MS/MS (89). Glycolysis was associated with treatment resistance in different type of cancers, thereby serving as a promising target of antitumor therapy (90). These studies suggested that the mechanisms of radiation resistance regulation were really complex and the combination of various proteomic techniques can be used to uncover novel radiation resistance biomarkers.

### 3.4 Extracranial Tumors in Children

Neuroblastoma was the most common extracranial solid tumor in children, with approximately 60% of children experiencing disease progression and metastasis (91). Abou-Antoun et al. compared the protein expression profiles of anchorage dependent and anchorage independent murine neuroblastoma and found that a dose of 2.5 Gy irradiation induced overexpression of survivin, Cyclin-dependent kinase 1 (CDC2), and Poly(ADP-Ribose) Polymerase 1 (PARP1) in unanchored neuroblastoma, and knockdown of survivin combined with multitargeted receptor tyrosine kinase inhibitor sunitinib enhanced the radiosensitivity of anchorage independent neuroblastoma; Anchorage-independent neuroblastoma has a different protein expression profile when compared with anchorage-dependent tumors and thus exhibits a higher degree of malignant phenotype and much more resistance to radiotherapy (92). CDC2 played a crucial role in regulating the transition of cells from G2 phase to mitotic M phase, targeting CDC2 lead to cell cycle arrest at the G2 phase (93). Additionally, PARP1 was a common nuclear protein and played an essential role in the stabilization of DNA replication forks; suppressing PARP1 was a complementary to conventional therapy by broken DNA repair mechanisms (94). Overall, these results suggested that inhibition of these altered proteins and their related signaling pathways has the potential to improve radiosensitivity of extracranial tumors.

## 4 Challenges of Proteomics

**Figure 3:**
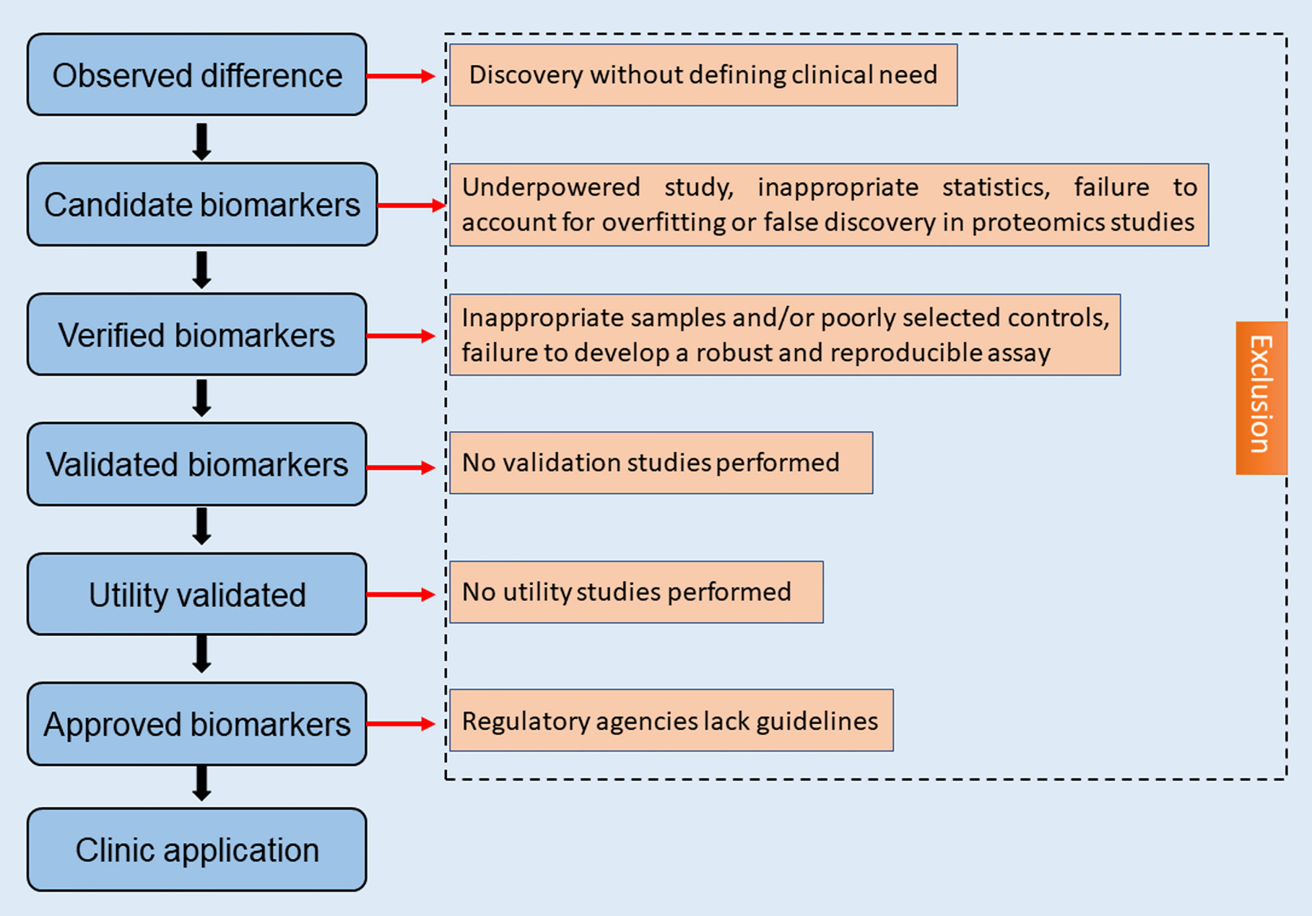
Radiosensitivity biomarkers development from proteomics to clinical.

Although proteomics focusing on the expression altered proteins and their activities in cells and organisms, this technology was still in its initial stage. There were many problems to be solved: firstly, sample preparing process, there was no uniform experimental conditions yet secondly, although a large number of proteins related to radiosensitivity have been discovered using proteomics techniques, more effective methods were required to identify the most specific targets (95). Moreover, the current proteomics analysis was expensive, which limits the possibility of further expanding the sample size; the detailed molecular mechanisms of screened biomarkers have not been well clarified; lastly, there are a great amount of work need to do for the application of radiosensitivity biomarkers that developed from proteomics to clinical (**Figure 3**). It was believed that with the application of new technologies, large-scale proteomics analysis will become possible.

## 5 Summary and Perspective

The use of proteomics to analyze radiation sensitivity biomarkers in tissues and plasma of cancer patients treated with radiotherapy has shown great promise and could clear some of the obstacles in precision tumor therapy. With the development of high-precision mass spectrometer, comprehensive analysis software and multi-model database, it was possible to comprehensively analysis the mechanisms underlying radiation resistance. This was helpful in the screening of patients appropriate for receiving radiotherapy in clinical practice, and to design novel targeted agents based on the discovered biomarkers, thus guiding the implementation of precision radiotherapy.

## Author Contributions

HL and HG contributed equally to the study. All authors contributed to the article and approved the submitted version.

## Conflict of Interest

The authors declare that the research was conducted in the absence of any commercial or financial relationships that could be construed as a potential conflict of interest.

## Publisher’s Note

All claims expressed in this article are solely those of the authors and do not necessarily represent those of their affiliated organizations, or those of the publisher, the editors and the reviewers. Any product that may be evaluated in this article, or claim that may be made by its manufacturer, is not guaranteed or endorsed by the publisher.
